# Foetal Intrapartum Compromise at Term: Could COVID-19 Infection Be Involved? A Case Report

**DOI:** 10.3390/medicina59030552

**Published:** 2023-03-11

**Authors:** Adrian-Ioan Toma, Bashar Haj Hamoud, Caliopia Gavril-Parfene, Mihaela Farcaş, Romina-Marina Sima, Liana Ples

**Affiliations:** 1 Neonatology Department, Life Memorial Hospital, 031593 Bucharest, Romania; 2 Faculty of Medicine, University “Titu Maiorescu”, 040441 Bucharest, Romania; 3 Department for Gynecology, Obstetrics and Reproductive Medicine, Saarland University Hospital, Kirrberger Straße 100, Building 9, 66421 Homburg, Germany; 4 Obstetrics–Gynecology Department, Life Memorial Hospital, 010719 Bucharest, Romania; 5 Department of Obstetrics and Gynecology, Carol Davila University of Medicine and Pharmacy, 050474 Bucharest, Romania

**Keywords:** COVID-19, placental histology changes, foetal hypoxia, therapeutic hypothermia, cooling

## Abstract

The impact of the SARS-CoV-2 infection on pregnancy has been studied and many reports have been published, mainly focussing on complications and in utero transmission with neonatal consequences. Although the effects of other viruses on foetuses are well known, the impact of maternal COVID-19 during pregnancy is not completely understood. We report a case of acute foetal intrapartum hypoxia without other risk factors than maternal COVID-19 disease 2 weeks previous to birth at term. Placental histological changes suggested that the viral infection could have been the culprit for the unfavourable outcome during labour. The neonate was promptly delivered by Caesarean section. Neonatal intensive care was started, including therapeutic hypothermia. The procedure was successful, the evolution of the neonate was favourable, and she was discharged after 10 days. Follow-up at 2 months of life indicated a normal neurological development but a drop in head growth. The case raises the idea that pregnancies with even mild COVID-19 symptoms may represent the cause of neonate compromise in a low-risk pregnancy. An important follow-up in the neonatal period and infancy is required to identify and treat any subsequent conditions. Further long-term studies are necessary to identify a cause–effect relationship between COVID-19 pregnancies and the whole spectrum of neonatal and infant consequences.

## 1. Introduction

Since the beginning of the COVID-19 pandemic in 2019, millions of pregnancies have been exposed to the disease. The impact of the SARS-CoV-2 infection on the mother, placenta and foetus has been studied and many papers have been published, mainly focussing on pregnancy complications and in utero transmission with neonatal consequences [1]. The influence of viruses on pregnancy and the foetus is well known for many entities, such as Citomegalovirus (CMV), rubella, Zika, parvo 19, etc., but as the data from the pandemic accumulated, more and surprising consequences of SARS-CoV-2 in foetuses and neonates were found, although the initial studies did not find a significant impact [2]. The most widely reported COVID-19 associated morbidity in pregnancy was premature birth (OR-4.29) [3,4].

We report a case of foetal acute intrapartum hypoxia after maternal exposure to COVID-19 prior to delivery, with a good postnatal outcome due to prompt foetal extraction and neonatal intervention to limit the neurological consequences of hypoxia.

## 2. Case Report

### 2.1. Prenatal History

Here, we report a case of a 34-year-old primigravida with a low-risk uneventful pregnancy who had been infected with SARS-CoV-2 at 37 weeks of gestation. The patient received two doses of the Pfizer BioNTech vaccine prior to pregnancy. The symptoms were mild with rhinorrhoea and headache for a couple of days and no fever, and COVID-19 was confirmed by a SARS-CoV-2 RT–PCR test. The blood tests performed according to the hospital’s policy revealed no inflammatory status, normal C reactive protein (CRP), and a normal blood count. Anti-nucleocapsid antibody tests performed during her hospital stay were positive (3.91 Um, CMIA Architect Abbott method with a 1.40 index). SARS-CoV-2 anti-spike antibodies were found (1653.31 AU/mL by the same method, with a cut-off of 50 AU/mL). Vaginal swabs were negative for pathogens. In the first trimester, the patient had low risk of FGR and pre-eclampsia (1/307 for FGR and 1/2541 for pre-eclampsia) and the ultrasound scans revealed normal foetal growth. 

At 39 + 1 weeks, the patient went into labour, which progressed normally until 6 cm cervical dilatation, when uterine tachysystole occurred and the foetus became moderately tachycardic (182 bpm). Tocolytic measures were taken, and the mother received mask oxygenation to improve foetal status. At 8 cm dilation, membrane rupture revealed thick meconial stained liquid and the foetus heart rate soared to 196 bpm. An emergency Caesarean section was decided because of the uncertain foetal status and a female baby weighting 3380 g was delivered with an Apgar score of 7/8. The placenta was sent for a pathological examination to elucidate the poor foetal outcome. 

The mother’s postoperative evolution was good. A monochorionic monoamniotic placenta was examined, which weighed 540 g and had a diameter of 16.5 cm and a thickness of 2 cm, had thin, semi-transparent membranes, an umbilical cord with a central insertion, was tri-vascular with a gelatinous consistency, and had a foetal surface with prominent vessels and a maternal surface consisting of friable, pale grey cotyledons. 

**Figure 1:**
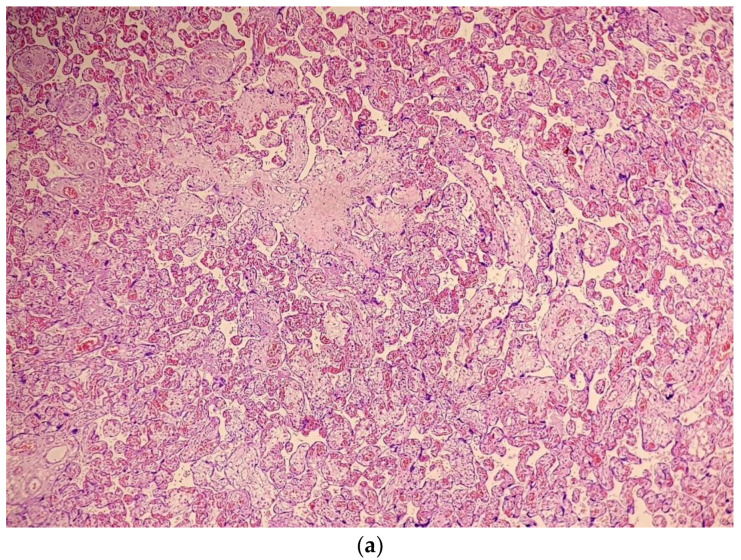

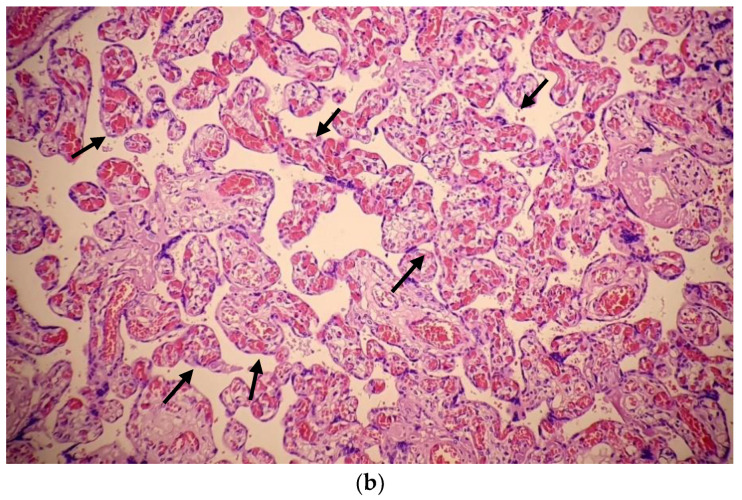
(**a**): Placenta with diffuse chorioangiosis (×4); (**b**): Important vascular changes of the terminal chorionic villi consistent with chorioangiosis (black arrows) (×10).

**Figure 2:**
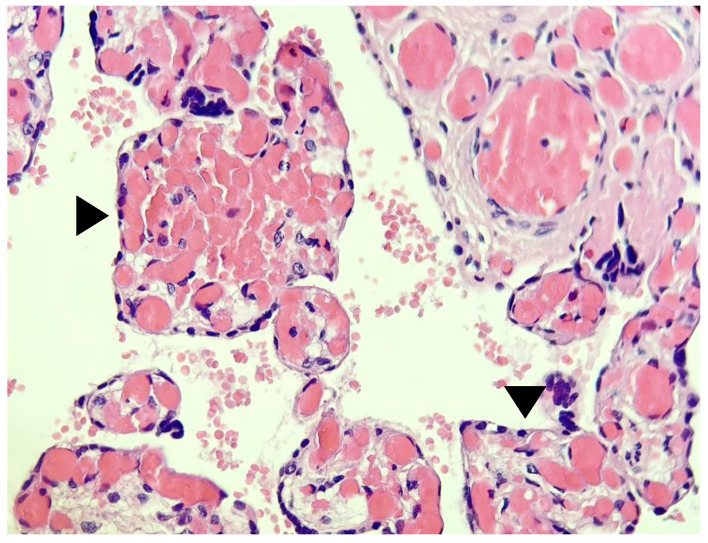
Detailed image showing >10 capillaries per terminal chorionic villus (black arrowhead) (20×).

**Figure 3:**
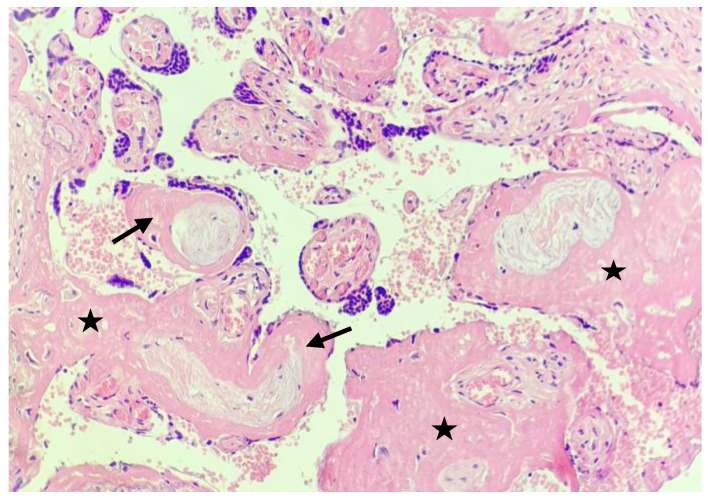
Detailed image showing marked peri-villous (star) and intra-villous (black arrow) fibrin deposition with dystrophy and ischaemic changes 󠅢(20×).

The examination of the placenta revealed COVID-19 related changes consisting mainly in chorioangiosis (Figure 1 and Figure 2), and important intra-villous and peri-villous fibrin deposition (Figure 3).

### 2.2. Neonatal Course

The neonate needed bag and mask ventilation at delivery. She was then intubated and mechanically ventilated due to deterioration of her respiratory status.

The arterial cord blood gas values were pH 7.10, PaCO_2_ 52.8 and mmHg base excess −12 mEq/dl. The modified Sarnat examination [5] at 1 and 3 h did not fulfil the criteria for the initiation of therapeutic hypothermia (cooling), as two items from the moderate category column were present, but at the examination at 5 h, she presented with three items from the column for the moderate encephalopathy stage (level of consciousness: lethargic; tone: mild hypotonia; absent primitive reflexes). Therefore, as the criteria had been met (perinatal distress, need for resuscitation at delivery, modified cord blood gases and modified clinical examination) [6,7], cooling was initiated at 5 h and 30 min, according to the internal protocol of the unit.

We used a Blanketrol III cooling mattress and maintained the core temperature of the neonate at 33.5 °C for 72 h. Rewarming was initiated after 72 h of cooling, with a rate of 0.5 °C/h for 6 h, and this occurred without complications.

A head ultrasound was performed before the initiation of cooling to exclude antenatal pathologies or malformations [8]. There were no identified antenatal haemorrhages, ischemia or malformations.

The patient was mechanically ventilated for 48 h. The ventilation parameters were modified according to the blood gases using the pH-stat approach [9,10]. She was extubated at 48 h of life and maintained at normal oxygen saturation and normal PaO_2_ and PaCO_2_ values in ambient air. 

During cooling, her blood pressure was maintained within normal values. The patient received dopamine at 5 mg/kg/min during the first day of her life, then, due to a normal blood pressure, the dopamine infusion was stopped. The blood pressure was maintained within normal limits during rewarming. 

Blood glucose and electrolytes were monitored both during cooling and rewarming, and were between normal limits.

We used blood lactate dehydrogenase (LDH) [11,12], creatine-kinase (CK) and creatine-kinase muscle-brain (CK-MB) [13] as markers of tissue hypoxia. LDH was increased during the first 3 days of life, but the values were under the cut-off value for risk of severe prognosis (1054 U/L) [12]. The values of LDH decreased within normal limits from Day 4 of life onward. CK and CK-MB increased during the first 3 days and then decreased to normal on Day 5 of life. Two transfusions of fresh frozen plasma were administered on Days 1 and 2 of life. 

**Figure 4:**
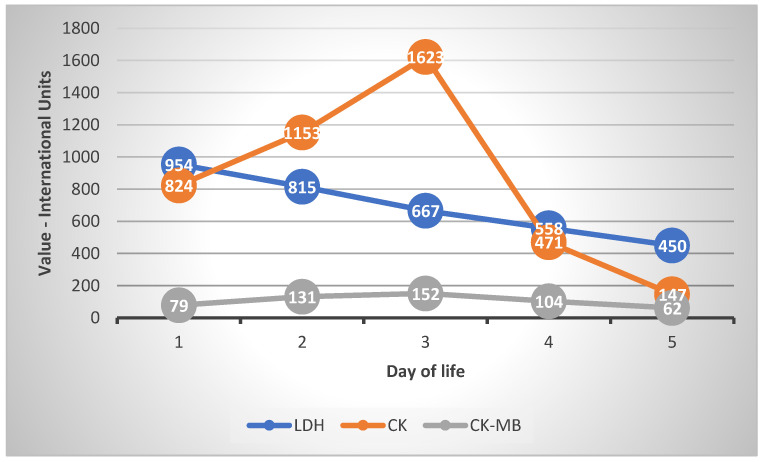
Boold markers of asphyxia.

The evolution of the markers can be followed in Figure 4

At the end of the first week of life, the patient presented with several subcutaneous nodules on the upper back, suggestive of subcutaneous fat necrosis associated with an increased value of both total and ionized blood calcium [14]. The nodules were monitored clinically, and involution was noted during the first month of life. According to the guidelines [14], calcium and Vitamin D were monitored, and the administration of Vitamin D was postponed until normalisation of the calcium and Vitamin D values at 1 month of life. 

Regarding the SARS-CoV-2 status, the neonate tested negative for IgG anti-nucleocapsids and presented with a titre of 4506.26 AU/mL for the anti-spike IgG antibodies (no active infection, antibodies passively transferred post-vaccination [15].

**Figure 5:**
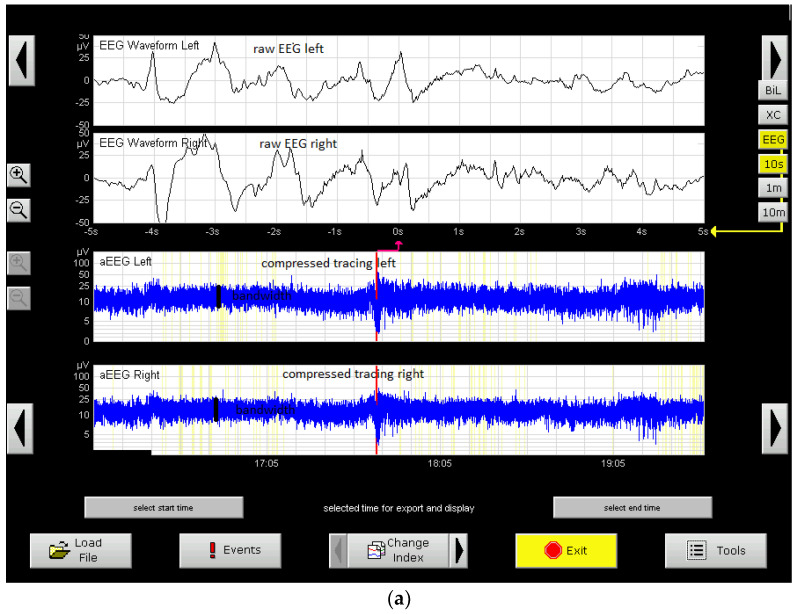

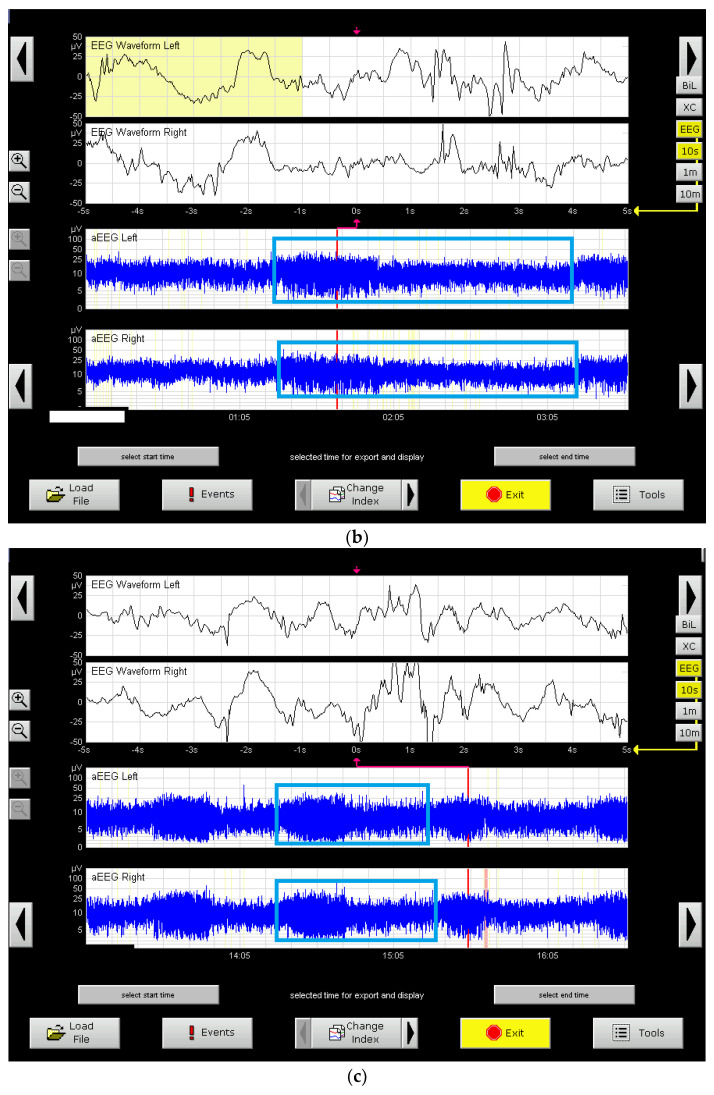
(**a**) aEEG monitoring. Sample tracings from 6 to 96 h of life. The upper 2 channels represent raw EEG-C3-P3; C4-P4 (raw tracing left and right on the figure). The lower channels (blue) represent amplitude integrated EEG (aEEG) (compressed tracing). The amplitude of both tracings is measured in microvolts-(µV)-scale figured at the left of the tracing. Tracing at the beginning of the hypothermia-cerebral activity present, but a narrow band-width (black line in both lower-compressed tracings). This is to be compared to the two lower channels in (**b**,**c**), where the bandwidth is larger. (The significance of this finding is that the bandwidth is proportional to the number of neurons that are active and discharge impulses-a narrow bandwidth in the beginning signifies a lower number of active neurons due to asphyxia). (**b**) aEEG monitoring. Tracing at 48 h. Emerging sleep-wake cycles are noted on the compressed tracing (lower two channels-see above)-framed in light blue on the figure-with an enlarged bandwidth portion-representing a quiet sleep interval and a narrower bandwidth segment representing Rapid Eye Movement (REM) sleep or awake interval. The cycle is not so regular and with approximately equal segments to the normal sleep–wake cycle in (**c**). (**c**) aEEG monitoring of the patient-same channels as (**a**). Continuous normal voltage pattern. In the lower tracings, that represent the aEEG compressed tracing, a normal sleep–wake pattern could be noticed-framed tracing-the first half of the framed tracing represents the quiet sleep pattern with discontinuous activity-the larger bandwidth, the second half-the REM sleep or wakefulness-decreased bandwidth, more continuous tracing.

Amplitude integrated Electroencephalogram(aEEG) monitoring was used during cooling and 24 h after rewarming. This was performed with a BRAINZ II device. The traces showed that cerebral activity was initially present with a narrowed bandwidth during the first 48 h of life (Figure 5a). At 48 h, emerging sleep–wake cycles [16] were noticed (Figure 5b). The pattern then became a continuous normal voltage (CNV) after 48 h of life and remained at CNV during and after rewarming with normal sleep–wake cycles pattern (Figure 5c). Seizures were not detected on the traces.

The whole purpose of aEEG two channel monitoring is to compare the electrical activity in the two hemispheres. The raw tracings are represented by C3-P3 on the left side and C4-P4 on the right. According to the 10–20 system regulations, the electrodes on the left have uneven numbers and the electrodes on the right have even numbers. C comes from Central and P from Parietal and those electrodes have a unique position described by a 10–20 system. 

The left and right examples are shown on the aEEG tracings in Figure 5a-c.

Considering all this, the reading of both raw and compressed channels showed no difference between the regions of the left and right hemispheres that were monitored. So, as expected, there is no difference between the left and right tracings-waveforms, amplitudes, frequencies in the raw tracing, amplitude, pattern and bandwidth in the compressed tracing. 

**Figure 6:**
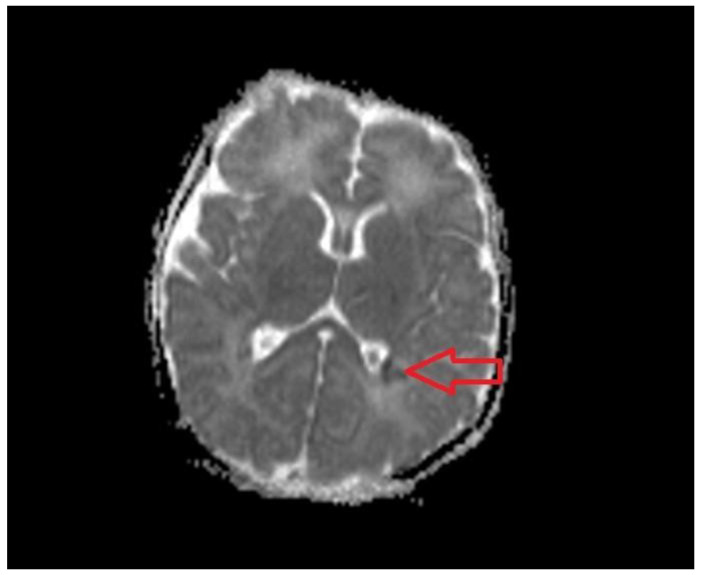
MRI on Day 5. ADC map: white matter lesion, left (shown by the arrow).

A head Magnetic Resonance Imaging (MRI) examination was performed on Day 5 of life. The examination identified a punctate white matter lesion pattern [17] adjacent to the occipital horns of the lateral ventricles (Figure 6).

The patient was discharged from the hospital at 10 days of life. 

An ophthalmologic examination was performed at 2 weeks of life and was within normal limits with no eye deviation and a normal funduscopic examination.

The neurologic examination at discharge was performed according to the Amiel-Tison neurological examination of the new-born [18,19]. No anomalies of passive tone or reflexes were detected. During the “pull to sit” manoeuvre, the control of the head was considered “too good”, which is a sight sign of hypertonicity. The head circumference at delivery was 34.5 cm; the head circumference at discharge was 35.5 cm. The hearing screening was normal.

According to its definition, neonatal encephalopathy is associated with depressed consciousness, difficulties in initiating and maintaining respirations, abnormal tone and seizures [20]. The consequences of hypoxic Ischaemic Neonatal Encephalopathy, mainly cerebral palsy, are characterised (among other features) by abnormalities of the tone [21]. The examination used in this case was the “Amiel Tison” neurologic examination of the newborn [22]. This examination assesses the general status of the neonate, the relationship with the environment, movements, the active and the passive tone and reflexes [22]. The active tone is defined here as the contractions of a certain group of muscles as a response to a stimulus, movement, in order to maintain the position of the body. The main manoeuvre to elicit an active tone response is the ”pull to sit” manoeuvre, In which the head is lifted from the surface by pulling, and the shoulders with both hands. This assesses the response of the flexors and extensors of the neck. The neck muscles are the first muscles to become (according to Amiel Tison) under the voluntary control of the pyramidal/corticospinal system. Therefore, the answer to this manoeuvre will be a contraction of the extensors and flexors of the neck and maintenance of the head in a vertical position for a few seconds. After pulling to sit, the manoeuvre is repeated backwards and the head should be maintained in the axis of the body by the same contraction until it reaches the horizontal. The abnormal results of this manoeuvre could be, in the case of a decreased tone (hypotonia), the passive movement of the head from back to front during pull to sit and from front to back during the opposite manoeuvre, in the absence of contractions of the muscles. In the opposite situation, there will be sustained contraction of the extensors and the head will point backwards during the pull to sit manoeuvre and will remain so during the pull back to horizontal. This would signify hypertonicity (increased tone). In our case, the baby attained control of the head during the pull to sit manoeuvre, but the examiner noted that the tone of the extensors was higher than normal (by a few seconds) so it was scored as a sign of mild hypertonicity which—as mentioned above—represents both a marker of neonatal encephalopathy and a tone abnormality that could be related to a future risk of cerebral palsy. Beyond the above (relation between tone abnormalities and diagnosis and future risks, the relevance of this finding is that, because of the risk factors and the tone problems, the neonate should benefit from an early intervention programme in physio-kineto-therapy and rehabilitation [22].

The neurological follow-up examinations were performed according to the Amiel–Tison examination for the infant and child [18], and showed no abnormalities of the active tone, passive tone or reflexes. At 2 months, the baby had good head control during the “pull to sit” manoeuvre, raised the head in prone position, and there were no motor deficits.

## 3. Discussion

The impact of COVID-19 on pregnancy outcome is now well established, and complications such as pre-eclampsia, intrauterine growth restriction and premature birth (spontaneous or iatrogenic) have been proven by many studies. In those studies, neonatal status was also correlated with disease severity, gestational age and maternal complications, considering that in utero transmission of the virus is rare [1,2,3]. Less is known, however, about the specific placental changes caused by the presence of the virus or inflammatory status. Even fewer data are available on the direct or indirect impacts of the virus on neonates.

Considering that the pregnancy was low risk for late FGR and had no previous sign of placental insufficiency until the COVID-19 episode, we postulate that the placenta acted as a gatekeeper for the virus, protecting the foetus from infection, but also underwent pathological changes consisting of chorioangiosis, villi atrophia, and extensive peri-villous and extracellular fibrin deposits. Our case suggests that even mild disease can induce placental damage and affect its adaptations and those of the foetus to the conditions of labour.

It has already been proven that the means of entry of SARS-CoV-2 entry to, and spread through, the placenta is via the main receptors: the angiotensin-converting enzyme 2 (ACE2) receptor [23] and the serine protease TMPRSS2 [24]. Other authors considered that the low expression of both receptors at the same time and the lack of evidence for those receptors in the chorio-amniotic membranes in the third trimester (unlike other viruses such as CMV, Zika, etc.) can explain why the virus does not affect the foetus and why the placenta acts as a gatekeeper [25]. In response to the virus in the placenta, natural killer cells and cytotoxic T cells are activated and destroy the cells that are already infected. Moreover, production of antibodies will eliminate some viral particles, but both defence mechanisms can be double-edged: tissue damage and antibodies captured by the Fc receptor target cells found in the placenta can have deleterious effects on the placental morphology and function [26,27].

A mechanism through which both the placenta and foetus can be affected is related to the severe cytokine storm that can intervene through endothelial and complementary dysregulation [28].

There are studies reporting that viruses do not have to cross the placenta in order to affect foetal brain development and the response to hypoxic challenges [29]. In our case, the mild form of COVID-19 expressed by the mother excluded that mechanism (i.e., vertical transmission) and placental changes were left as the only factor responsible for the foetal hypoxia.

Many studies have stated that an impaired placenta will lead to maternal mal-perfusion and affect the exchange of gas and nutrients [30,31]. The impairment is increased during labour by the rise in intrauterine pressure up to 70 mmHg and leads to a consecutive reduction in utero–placental perfusion and to intermittent placental hypoxia [32]. The foetus can cope with intermittent, though marked, reductions in oxygenation during contractions, unless her/his mechanism of compensation is affected (as in Fetal Growth Restriction (FGR)) or there is a suboptimal gas exchange caused by placental impairment and maternal mal-perfusion [33,34]. In our case, placental failure was caused by extensive fibrin deposits, and thrombosis of the villi could have acted as a major cause of the impaired foetal oxygenation and the onset of hypoxia.

Another condition that worsened the placenta–foetal gas exchange was uterine hypercontractility, which occurred suddenly after a normally evolving labour. The literature is consistent in demonstrating that uterine tachysystole gives no time for placental reperfusion and foetal recovery [13]. There is a lack of evidence as to whether COVID-19 infections can also affect the uterine muscle and interfere with normal contractility in labour. We previously reported a case of affected uterine contractility in a COVID-19-associated pregnancy in which histologically significant changes indicating ischemia were found [35].

We found another two case reports of neonatal asphyxia in the literature: one with severe brain damage after a mild disease episode in the early second trimester [36] and another in an asymptomatic mother whose baby was extracted because of severe distress and developed multiple organ failure with a difficult evolution [37]. As in our case, the authors postulated that the poor outcome was caused by extensive histological changes in the placenta caused by the inflammation produced by SARS-CoV-2.

There seem to be more changes and hallmarks of COVID-19-affected placentas. Some authors observed signs of maternal vascular mal-perfusion, extensive fibrin deposits, inter-villous thrombi and an increased incidence of chorangiosis as a sign of chronic hypoxia [38]. Others suggested that a prominent B lymphocytes infiltration might be a histopathological hallmark that differentiates the histiocytic inter-villositis of SARS-CoV-2 infection from chronic histiocytic inter-villositis of unknown origin [39]. The histological examination of the placenta, in our case, was also consistent with the findings in the literature, confirming the impact that the virus had on it, and explaining the insufficient adaptation and oxygenation during labour that led to foetal hypoxia.

The impact of COVID-19 on poor foetal and neonatal outcomes, even in mild or asymptomatic forms, was also reported by Jaiswal, who suggested that the effects are not immediate in all cases, and the offspring of the COVID-19-affected mothers are at risk for later neurological and psychological pathological issues [40].

From the neonatal point of view, this was a case of moderate perinatal asphyxia, according to the Sarnat stages [5,6,12,19]. The decision to treat the neonate with whole-body hypothermia was based on the evolution of the patient. Even if, in the first 3 h of life, the patient did not meet the criteria for cooling [2,3,20], the neurological exam between 3 and 6 h showed anomalies in the moderate encephalopathy column that corroborated the evidence of perinatal insult (foetal distress, meconium-stained amniotic fluid and the need for resuscitation at delivery), and anomalies of the blood gases from the umbilical cord represented criteria for start therapeutic hypothermia [5,6,41].

As complications during and after the treatment, abnormalities of coagulation and subcutaneous fat necrosis were noted. These both represent consequences of asphyxia and of hypothermia, resulting in death of the brown fat cells [14]. The abnormality resolved after restriction of the use of Vitamin D during the first month of life and avoidance of supplementary calcium, except for breastmilk [42].

The lesion present in this case was identified in the MRI on Day 5 and consisted of punctate white matter injury in the periventricular area, also called a “focal/multifocal injury pattern” [43]. This pattern has been described in cases of milder asphyxia and circumstances leading to hypoxia without significant acidosis [44]. The lesion has a good prognostic value, as the patients with this lesion have either a normal outcome or milder and nonspecific deficits [17].

Together with MRI scanning, aEEG is considered to have very good prognostic significance [16]. Three items were found to have prognostic value: the background pattern and its evolution, the appearance of sleep–wake cycles, and seizures (the best predictor is the background pattern) [16]. In this case, the background pattern during the first 48 h of life did not consist of one of those factors associated with a bad prognosis (continuous low voltage, burst suppression or a flat trace) [45]. The evolution of the background pattern is also considered to have a good prognostic significance [14,25]. In this case, the normal background pattern (continuous normal voltage) appeared before 48 h of life; this occurrence has a very good prognostic value for a normal neurologic outcome in cases of patients treated with hypothermia [46]. Another important pattern to look for is that of cycles (sleep–wake cycles). The appearance of this pattern before 36 h of life is associated with a good prognosis [16,47]. In this case, the pattern appeared before 48 h but after 36 h. However, according to other studies, in the case of therapeutic hypothermia, the appearance of the sleep–wake cycles is a little delayed [43,44], so this could also be considered a good prognostic sign.

We observed that the follow-up of the new-born was in accordance with the literature, which now contains an increasing number of reports about neonates born to mothers with SARS-CoV-2 infection and the augmented concerns regarding the long-term impact on infants [48,49]. The milder maternal course (and even the neonatal course) was probably related to the previous vaccination, which is also strongly supported by other publications [50].

The histological changes induced by SARS-CoV-2 in the placenta have already been recognized by many studies. Recent studies [37] have demonstrated that placental impairment can be found even in mild maternal disease. In a study from 2021, Jaiswal et al. found that placental injury consisting of foetal–maternal mal-perfusion, chorioangiosis, thrombosis of the foetal chorionic plate, fibrin deposition and other ischemic markers could be found at the microscopic level even in otherwise asymptomatic or mildly symptomatic SARS-CoV-2-positive pregnant women [40]. They stated that the consequences of these injuries, as far as the foetus is concerned, need to be investigated in extended studies.

On the other hand, even in uninfected placentas, ischemic injuries are responsible for poor labour tolerance in normal full-term foetuses. In an extensive expert review, Turner et al. detailed the mechanisms that are responsible for foetal intrapartum hypoxia in normal full-term babies when placental mal-perfusion caused by various factors (inadequate trophoblastic invasion, inflammation, placental abnormalities) negatively impact the foetal oxygenation status. The authors demonstrated that during labour contractions, at the placenta level, there is a repeated sequence of ischemia and reperfusion that will determine a significant reduction in proangiogenic factors, activate the inflammatory cytokine pathways and reduce foetal capillary perfusion, placing the foetus at a risk of inadequate gas exchange and thus to hypoxia. The authors considered that placental dysfunction can lead to foetal compromise as a gradual process by affecting the foetal myocardial capacity to respond to the peripheral chemoreflex due to a low glycogen store before labour, which is also caused by placental dysfunction [30].

We consider that, in our case, this could also be the mechanism that triggered foetal hypoxia in labour. We excluded placental dysfunction prior to COVID-19 on the basis of the normal foetal growth, the normal amniotic liquid and the normal uterine and foetal Doppler indexes. Moreover, the pregnancy benefitted from risk assessments in the first trimester for restricted growth and pre-eclampsia, and was labelled as low-risk. The labour had a normal onset and evolution, and the appearance of the hypoxic signs (foetal tachycardia, meconium-stained amniotic liquid) was promptly relieved by Caesarean section. The parameters of neonatal acidosis were mild but can be explained by the short period in between the onset of hypoxia and resolution of the birth.

Examination of the placenta did not show macroscopic chronic ischemic signs such as extended infarction or calcar deposits, but the microscopic examination indicated injuries that may have been responsible for the poor response to intrapartum ischemic stress, as explained above. There are also other studies that have reported brain injuries or poor neonatal outcomes related to COVID-19 infections, including some asymptomatic or mild cases, and attributed the outcome to the histological changes in the placenta induced by SARS-CoV-2 [4,35,46,47,48].

## 4. Conclusions

The case we reported is of unexpected neonatal hypoxia without other risk factors than a recent mild maternal COVID-19 disease. The neonatal outcome was excellent because of prompt diagnosis and extraction by Caesarean section, followed by intensive neonatal care, including a cooling procedure. Evidence is accumulating to show that SARS-CoV-2 in pregnancy can have severe neonatal consequences, mainly caused by placental impairment and poor hypo-oxygenation tolerance during labour by both the foetus and the placenta. Further studies and reports on similar cases may highlight the need for close surveillance during pregnancy and the neonatal period, and long-term follow up to prevent future neurological and psychiatric disorders in the affected babies.

## Data Availability

The data presented in this study are available on request from the corresponding author.
